# Supplementary material for: Perspectives on preconception care in Ethiopia: Social, cultural, and structural determinants
Source: PLoS One. 2026 Jun 5;21(6):e0351142. doi: 10.1371/journal.pone.0351142 (PMC13240867; doi:10.1371/journal.pone.0351142)
Supplement: S1 File — (DOCX) [file pone.0351142.s001.docx]

**Semi-Structured Interview Guide: Perspectives, Experiences, and Perceptions of Pre-Pregnancy Care Among Reproductive-Age Individuals**

**Section 1: Perspectives and Perceptions of Pre-Pregnancy Care**

- What are your perspectives or perceptions about pre-pregnancy care for women or couples?
- What had you heard or understood about pre-pregnancy care before this study?
- How would you describe your current understanding and perception of pre-pregnancy care?
- Which aspects of pre-pregnancy care are you familiar with or have formed opinions about?
- Who do you think should receive pre-pregnancy care?
- When do you think pre-pregnancy care should begin?
- Where do people in your community usually seek pre-pregnancy care services?
- How have your experiences and interactions influenced your understanding of pre-pregnancy care?
- How do discussions with your partner, family, or community shape your perceptions of pre-pregnancy care?
- Are there gender roles or expectations that influence how people perceive or engage with pre-pregnancy care?
- How has your experience with healthcare services shaped your perception of pre-pregnancy care?
- What efforts have you observed from the health system to improve awareness or understanding of pre-pregnancy care?
- How do healthcare services address the needs of different groups in providing pre-pregnancy care?

**Section 2: Attitudes, Perspectives, and Experiences Toward Pre-Pregnancy Care**

- What are your personal views or perceptions about the importance of pre-pregnancy care?
- Based on your experience, what benefits do you associate with pre-pregnancy care?
- How do your beliefs and values shape your attitudes and perceptions toward pre-pregnancy care?
- What are the common perceptions or attitudes toward pre-pregnancy care in your community?
- How do cultural norms and societal expectations influence your views and experiences?
- How do influential community members (e.g., elders) shape people’s perceptions of pre-pregnancy care?
- How do your experiences with healthcare services influence your attitudes toward seeking pre-pregnancy care?
- How do healthcare providers’ attitudes shape your willingness to use these services?

**Section 3: Experiences and Practices Related to Pre-Pregnancy Care**

- Can you describe your experiences with pre-pregnancy care services?
- What actions or practices do you engage in when thinking about pregnancy?
- How do your experiences align with or differ from community expectations?
- What do women in your community typically do before becoming pregnant?
- How do family or community expectations influence your experiences and practices?
- Why do you think some people are aware of pre-pregnancy care but do not use it?
- How do men in your community participate in or support pre-pregnancy care?
- Who usually makes decisions about seeking pre-pregnancy care in households?
- How do social issues (e.g., domestic violence) shape women’s experiences in accessing care?
- How supportive is your community toward pre-pregnancy care for both women and men?
- How have your experiences with health facilities influenced your access to pre-pregnancy care?
- What health system factors (e.g., location, service hours, transportation) shape your experience of accessing care?

**Section 4: Additional Perspectives and Experiences**

- What are your perspectives on the roles of men and women in pre-pregnancy care?
- How do these roles influence people’s experiences and perceptions of care?
- How do gender, socioeconomic status, ethnicity, or cultural background shape people’s experiences and perceptions of pre-pregnancy care?
- Can you share any experiences or challenges related to accessing care influenced by these factors?

**Section 5: Demographic Information**

Date:
Participant Study ID: ___________

1. Age:

- 18–29 years
- 30–39 years
- 40–49 years
- Prefer not to say

1. Gender:

- Men
- Women
- Other (Specify) __________
- Prefer not to say

1. Highest educational qualification:

- Primary school (≤ Grade 8)
- Some secondary (did not complete Grade 12)
- Completed secondary diploma
- College/trades/apprenticeship
- Undergraduate degree
- Graduate degree
- Prefer to specify __________

1. Marital/partner status:

- Single
- Married/common-law
- Widowed
- Divorced
- Separated
- Prefer to specify __________
