# Supplementary material for: Perspectives on preconception care in Ethiopia: Social, cultural, and structural determinants
Source: PLoS One. 2026 Jun 5;21(6):e0351142. doi: 10.1371/journal.pone.0351142 (PMC13240867; doi:10.1371/journal.pone.0351142)
Supplement: S3 File — (DOCX) [file pone.0351142.s003.docx]

| **Selective Codes** | **Axial Codes** | **Open code focus** | **Sample illustrative quotation** | **Participant** |
| --- | --- | --- | --- | --- |
| **1. Fragmented Knowledge Pathways and Practices across Reactive, Guided, and Constrained Routes** | Awareness and understanding of PCC | Misconception that PCC starts after pregnancy | *“Honestly, we usually think of the checkups after a woman is already pregnant… this idea of getting ready before, it is not clear to many of us”* | P05-F |
|  | Awareness and understanding of PCC | Recognition of male preconception health needs / PCC viewed as female-only care | *“I didn’t think this was something for me until problems [infertility] happened. It felt like it was only for women”* | P04-M |
|  | Information Sources | Peer storytelling and testimonies / family influence | *“After I learned, I told my cousins, they listen more to me than to clinics”* | P06-F |
|  | Information Sources | Media campaigns / social media | *“I first heard about it on TikTok and Facebook… in government places I’ve never seen it being talked about”* | P08-F |
|  | Self-Directed and Continuous Learning | Blending faith and medical information | *“God provides doctors and medicine”* | P04-M |
|  | Understanding and Purpose | Reliance on traditional or faith-based PCC practices / fatalism | *“They say, if God gives, you will have a child, so why prepare?”* | P17-F |
|  | Timing and Importance | Awareness of pregnancy loss and preventable risks | *“I had several pregnancy losses before, and because of that, I started visiting the hospital more often… I decided to follow their advice and started the follow-up to prepare my body better before trying again”* | P06-F |
|  | Gaps and Missed Opportunities | Wealth-based structural inequities / limited access | *“In government hospitals there is no such care, only in private if you can pay”* | P08-F |
|  | Gaps and Missed Opportunities | Missed early opportunities due to stigma | *“When I found out about these things, it was after my friend had a bad experience… unplanned pregnancy. We don’t talk about it openly because people judge you”* | P02-F |
|  | Discreet and Constrained Practices | Hidden visits / exclusion of unmarried women | *“They refused me to take birth pills… because I was not married”* | P06-F |
|  | Proactive and Supportive Practices | Male emotional/financial support | *“My husband sent me transport money right away… she said she felt respected and continued her visits”* | P09-F |
|  | Discreet and Constrained Practices | Men indirectly involved | *“I haven’t gone with her yet… it feels like the clinic is more for her, and we men are not expected to be there”* | P11-M |
|  | Reactive and Restrictive Practices | Unsafe use of pharmacy-based services / constrained informal practice | *“Some women face health problems later, like bleeding… Because no one explains, they just take it and hope it works. The woman suffers, while the pharmacy only cares about selling”* | P05-F |
|  | Practice Barriers | Poor staffing / weak continuity | *“You go today, and the provider is not there, so you return home”* | P07-F |
|  | Emotional Dimensions | Couple calm / emotional meaning | *“Maintaining calm at home”* | P12-M |
| **2.Preventive Orientation and Reassurance** | Aspirational Parenting and Life-Saving Purpose | Aspirational parenting/nation-building | *“If we take care early, we give birth to children who can help our country tomorrow.”* | P06-F |
|  | \|  \| \| --- \|  \| Reassurance and Readiness \| \| --- \| | Emotional reassurance and confidence | *“It gives peace of mind before pregnancy”* | P02-F |
|  | Collective and Shared Responsibility | Shared duty / men’s supportive role  Couple communication and joint decision-makin | *“We should both be responsible. It’s our child, not just hers”*  *“Joint decision-making prevents pregnancy complications”* | P15-M  P11-M |
|  | Positive Orientation and Acceptance | Male willingness to participate | *“These days, men like me want to go with our wives, we don’t see it as shame”* | P16-M |
|  | Preventive and Responsibility-Oriented Mindset | Early information despite exclusion  Faith and parenting as responsibility frames | *“Even if they deny us, we need information early so we don’t suffer later”*  *“God gives children, but He also gives* | P01-F  P13-F |
|  | Willingness to Learn | Preference for practical, simple messages | *Preference for practical, simple messages* | P10-M |
| **3. Emotional and Psychological Impact** | Loss, Learning, and Resilience | Grief following loss / renewed motivation | *“Only after I lost the baby did I hear about pre-pregnancy care”* | P06-F |
|  | Fear and Anxiety | Fear of social exposure / stigma | *“We don’t talk about it openly because people judge you”* | P07-F |
|  | Emotional Consequences of Silence | Institutional exclusion and isolation | *“They refused me because I was not married”* | P08-F |
|  | Emotional Consequences of Silence | Concealed suffering / relational burden | *“She lives with heavy pain… They cry at night because they want a different path, but the burden of the child keeps them there”* | P05-F |
|  | Supportive Buffers and Positive Outcomes | Couple calm and trust | *“Joint decision-making prevents pregnancy complications”* | P04-M |
|  | Supportive Buffers and Positive Outcomes | Reassurance and peace of mind | *“It gives peace of mind before pregnancy”* | P17-F |
|  | Ambivalence and Contradictions | Male emotional discomfort / ridicule | *“If you go for check-ups when you are not sick, people may laugh and say, ‘Why are you worrying like a woman?’”* | P16-M |
|  | Ambivalence and Contradictions | Hope and guilt coexisting | *“Even though the care helped me a lot, it gave me peace of mind and helped us prepare mentally, I still felt guilty, like maybe I had broken God’s rule”* | P08-F |
|  | Advocacy and Promotion Strategies | Inclusion of men and women / visibility | *“Make it simple, make it visible, and invite both women and men—otherwise, nothing will change”* | P02-F |
| **4. Advocacy, Shared Responsibility, and Gendered Cultural Barriers** | Cultural and Religious Frames | Marriage as prerequisite for legitimacy | *“They refused me because I was not married”* | P06-F |
|  | Community and Social Norms | Gossip and social policing | *“We don’t talk about it openly because people judge you”* | P08-F |
|  | Cultural and Religious Frames | Integration of faith and medicine | *“God provides doctors and medicine”* | P07-F |
|  | Cultural and Religious Frames | Fatalistic beliefs | *“If God gives, you will have a child, so why prepare?”* | P11-M |
|  | Stigma, Shame, and Blame | Unmarried women stigmatized | *“Unmarried girls were judged for seeking care…carelessness”* | P07-F |
|  | Gender and Decision-Making Dynamics | Economic and power imbalance / abandonment risk | *“If they get pregnant, the man may disappear. Then the girl is left alone, without support, and she never had the chance to get care”* | P07-F |
|  | Community and Social Norms | Leaders normalizing PCC | *“If leaders don’t say it, people think it is not important”* | P07-F |
|  | Community and Peer Influence | Male visibility shifting norms | *“When men go with their wives, it encourages others to do the same”* | P14-M |
|  | Education and Literacy | Respectful, practical communication / clinic visibility | *“Give it a clear name and a visible spot at the clinic… Use loudspeakers, schools, and mosques/churches to repeat one message: ‘Prepare early for a safer birth.’ Invite husbands. Use our language and simple words. Keep hours reliable. Fix ambulance issues and share one phone number that works”* | P06-F |
| **5. Education and Community Influence as Levers** | Trusted Information Sources | Media and community as transformative pathways | *“Together, these accounts show education, community influence, and media as pathways to transform PCC into a visible, collective, and normalized practice”* | P08-F |
|  | Economic and Geographic Barriers | Economic hardship and cost barriers | *“Because in the city, there are many pressures—rent, transport, food costs. If you earn something small, you prioritize surviving, not going to the clinic… people may laugh and say, ‘Why are you worrying like a woman?’”* | P14-M |
|  | Economic and Geographic Barriers | PCC as payment-dependent / elite care | *“In government hospitals, there is no such care, only in private if you can pay”* | P07-F |
|  | Healthcare Access and Quality | Long waits / absent providers | *“You go today, and the provider is not there, so you return home”* | P06-F |
|  | Healthcare Access and Quality | Unsafe pharmacy-based entry point | *“They give you medicine at the pharmacy, but no one tells you how to use it or why”* | P05-F |
| **6. Structural and Health System Inequities** | Accessibility and Systemic Readiness | Privacy-sensitive framing for youth | *“It should be presented as health, not permission for sex… explain it as ‘preparing your body for the future’”* | P14-M |
|  | Systemic and Institutional Influences | Weak awareness campaigns | *“We don’t hear about it in the community, and we don’t see campaigns like we do for family planning”* | P08-F |
|  | Healthcare Access and Quality | Need for visible PCC infrastructure | *“Even just having a clear sign that says ‘Pre-Pregnancy Care Here’ would make us ask”* | P09-F |
|  | Family and Peer Linkages | Family support and early encouragement | *“My sister-in-law once wanted to check her blood. She called her husband from the clinic, and he sent transport money right away. She felt respected and continued her visits”* | P06-F |
|  | Family and Peer Linkages | Weak support before visible pregnancy | *“Before pregnancy, support is weak because they don’t see a belly yet. Unmarried women get the least support due to shame and gossip”* | P17-F |
|  | Community and Leadership Linkages | Male visibility shifting community norms | *“When others see a husband involved, it gives them courage too”* | P16-M |
|  | Institutional and Service Linkages | Weak continuity / stock-outs undermining trust | *“I went twice to ask for vitamins before pregnancy; they said ‘no stock.’ After that, I stopped trying. When this happens to one person, five others also give up”* | P03-F |
| **7. Social and Institutional Linkages for Change** | Policy and Structural Linkages | Government accountability / tangible action | *“Not just workshops or posters. We need services we can see and touch… free or subsidized check-ups, youth-friendly corners, and respect at clinics. Real action means changing daily life, not just talking about plans”* | P16-M |
|  | Policy and Structural Linkages | PCC linked to collective development | *“If we take care early, we give birth to children who can help our country tomorrow”* | P06-F |
