# Supplementary material for: Perspectives on preconception care in Ethiopia: Social, cultural, and structural determinants
Source: PLoS One. 2026 Jun 5;21(6):e0351142. doi: 10.1371/journal.pone.0351142 (PMC13240867; doi:10.1371/journal.pone.0351142)
Supplement: Attachment — Submitted filename: Comments on PCC.pdf [file pone.0351142.s005.pdf]

Dear Editor,

I would like to provide some feedback on the manuscript titled "Understanding Reproductive-Age Individuals' Perspectives on Preconception Care in Ethiopia." Overall, the paper is well-conceived and presents valuable insights into the topic, but it does need minor revisions.

1. Title Clarity and Focus

- **Feedback**: The title effectively captures the essence of the study, but consider making it more concise by removing less critical phrases. For example, "Understanding Reproductive-Age Individuals' Perspectives on Preconception Care in Ethiopia" could be shortened to "Perspectives on Preconception Care in Ethiopia: Social, Cultural, and Structural Determinants." This focuses the reader's attention on the primary research topic.

2. Establishing Context

- **Feedback**: The background provides essential context, particularly regarding the introduction of the PCC guidelines. However, consider including a brief mention of the specific health outcomes or public health significance associated with improved PCC in Ethiopia. This might enhance the urgency and importance of the study.

3. Methodological Rigor

- **Feedback**: The methods section outlines an appropriate approach for this type of qualitative research. It may be beneficial to elaborate on the selection criteria for participants and the rationale for employing maximum-variation sampling. This detail could enhance the robustness of your methodology and reassure readers about the representativeness of the sample.

4. Thematic Insights

- **Feedback**: The results section highlights significant themes that demonstrate the complexity of PCC uptake. To strengthen this section, consider providing brief examples or quotes from participants to illustrate the themes, particularly regarding "marital gatekeeping" and "emotional negotiations.” Rich qualitative data enhances the credibility and relatability of the findings.

5. Actionable Conclusions

- **Feedback**: The conclusions successfully address the complexities of PCC practice in Ethiopia. To further improve, consider specifying actionable recommendations based on your findings, such as potential interventions for healthcare providers, community organizations, or policymakers. Explicitly connecting these recommendations to identified themes could provide a clearer pathway for implementing the PCC guidelines effectively.

Overall Impression

Your work presents valuable insights into preconception care in Ethiopia, addressing gaps in the current literature by focusing on social and cultural determinants. By refining the title and enhancing specific sections, you can communicate your findings more effectively and emphasize the need for comprehensive, context-aware strategies in PCC implementation.

- **What specific social and cultural factors significantly influence the acceptance and uptake of preconception care among different demographic groups in urban Ethiopia?**
- **How do gender norms and roles affect men’s involvement in preconception care, and what strategies can be implemented to encourage their active participation in this process?**
- **What barriers do married women face in accessing healthcare related to preconception care, and how can healthcare services be adapted to better meet their needs?**
