# Supplementary material for: Perspectives on preconception care in Ethiopia: Social, cultural, and structural determinants
Source: PLoS One. 2026 Jun 5;21(6):e0351142. doi: 10.1371/journal.pone.0351142 (PMC13240867; doi:10.1371/journal.pone.0351142)
Supplement: Attachment — Submitted filename: Review PLOS One.docx [file pone.0351142.s004.docx]

**Reviewer’s comment to the Author**

PLOS One

Understanding Reproductive-Age Individuals’ Perspectives on Preconception Care in Ethiopia: Social, Cultural, and Structural Determinants--Manuscript Draft- Manuscript Number: PONE-D-25-65367

Thank you for giving me a chance to review this qualitative research on preconception care.

**General comments**

1. The manuscript addresses important issues that could contribute to / have implications for healthcare services in Ethiopia. The manuscript is well written overall. The content, introduction, and discussion sections are clearly and effectively articulated. However, the Results section requires substantial revision and reanalysis. The themes and subthemes should be more clearly derived from / aligned with the specific research question(s).
2. The document does not include line numbers, which would be helpful for reviewers to refer to specific lines precisely.
3. Please provide/attach the interview guide (or topic guide) used in the study. This is important for assessing how the emerged categories and subcategories in the results relate to the questions asked.
4. Please ensure the manuscript matches the formatting guidelines of **PLOS ONE**

Title:

5. I suggested the title should be modified as…” Exploring Knowledge, Attitudes, and Practices Regarding Preconception Care Among Reproductive-Age Individuals in Ethiopia: An Interpretive Description Study Using the Socio-Ecological and Intersectional Models”

**Abstract:**

6. You said, in Ethiopia, PCC remains almost absent in everyday health practice; the pooled utilization of PCC in Ethiopia is 26%. “[The magnitude of preconception care utilization and associated factors among women in Ethiopia: systematic review and meta-analysis, 2024 | BMC Pregnancy and Childbirth](https://link.springer.com/article/10.1186/s12884-025-07394-6)”, can we say almost absent? Please justify.
7. Similarly, you mentioned again: “This study provides the first in-depth, urban Ethiopian analysis showing how adults actively negotiate preconception care (PCC) within intersecting gender, moral, and structural systems.” However, more than three published qualitative studies on preconception care already exist in Ethiopia. As a researcher, particularly a qualitative one, honesty is essential to maintain the trustworthiness and credibility of the study.
8. You employed maximum variation sampling, yet your study participants were drawn from the reproductive-age group (both women and men), which constitutes a relatively homogeneous population. Do you believe maximum variation sampling is appropriate for this study?
9. Is interpretive description design more dominant in exploratory studies

Or Explanatory studies? Make sure that you use the appropriate design for your research.

10. Are all the listed keywords scientifically appropriate and aligned with standard terminology? Please modify the words included in the keywords.

**Introduction:**

11. What is the primary objective of this study—to explore the knowledge, attitudes, and practices (KAP) regarding PCC, or to examine participants’ perspectives on PCC within socio-ecological and intersectional contexts? Please justify your response by referring to the study title and conclusion, as there appears to be a potential mismatch between the stated aim (KAP-focused) and the interpretive, contextual emphasis in the title and conclusion.

**Methods**

12. Please attach the written informed consent document.
13. The guides explored knowledge, attitudes, and practices regarding PCC; perceived benefits and barriers; gender and cultural dynamics; and experiences with healthcare providers.

**Result**

14. You mentioned that pilot tests were conducted for clarity and cultural appropriateness before use. Could you elaborate on the number of participants included in the pilot, and was this pilot data included in the formal analysis? Please describe this process in detail within the data collection section.
15. Five women who were planning to conceive were included. How were these women identified? What method or tool was used for their identification?
16. Detailed descriptions within each theme and subtheme are vital for rigorous analysis and reporting.
17. The sub-themes within the seven main themes should be rewritten to consolidate similar concepts.
18. Since the main purpose of using maximum variation sampling is to collect information from a heterogeneous participant group, the analysis should describe and compare the differences and similarities among participants within each theme and subtheme.
19. Please specify which conceptual framework or model (e.g., the socio-ecological model) guided this study. The results section should then discuss the main findings in relation to this chosen framework.
20. The overall results presentation seems shallow, incoherent, and vague. The data likely requires re-analysis, and the presentation of themes and subthemes needs to be updated accordingly.

**Discussion**

21. Is the word "must" commonly used to formulate recommendations for different actors based on the findings of a single study?
22. You state, "Policy: Ethiopia’s reproductive health policy should integrate PCC into public services with clear guidelines." However, a national preconception care guideline was developed in 2024. What is the intended implication of your recommendation, given this existing policy?

**Conclusion**

23. In your conclusion, you stated, “PCC should combine biomedical prevention with relational and gender-sensitive practice.” This statement is unclear. According to the Ethiopian PCC guideline, the definition of PCC already includes a bundle of interventions aimed at identifying and modifying biomedical, behavioral, and social risks to improve pregnancy outcomes. Why, then, does your conclusion suggest that PCC *should* combine biomedical prevention? The guideline already encompasses this approach.
24. Similarly, in your conclusion, you wrote, “Providers must engage men and women as clients and supporters while ensuring confidential, stigma-free access for adolescents and unmarried women.” According to the Ethiopian PCC guideline, couple-based services are included as a guiding principle. Your statement should more directly align with this principle of couple-centered care.
25. Therefore, your implications and conclusions should be updated to reflect the context of the Ethiopian national PCC guideline.
