# Supplementary material for: Perspectives on preconception care in Ethiopia: Social, cultural, and structural determinants
Source: PLoS One. 2026 Jun 5;21(6):e0351142. doi: 10.1371/journal.pone.0351142 (PMC13240867; doi:10.1371/journal.pone.0351142)
Supplement: Attachment — Submitted filename: Response to Reviewers.docx [file pone.0351142.s006.docx]

**Authors Response**

| **Editor and Reviewer Comments** | **Comments/ Revisions Made** |
| --- | --- |
| **Editor comment** | |
| **Journal Requirements** | |
| Paper provides good context/background for Please ensure that your manuscript meets PLOS ONE's style requirements, including those for file naming. The PLOS ONE style templates can be found at <https://journals.plos.org/plosone/s/file?id=wjVg/PLOSOne_formatting_sample_main_body.pdf> and <https://journals.plos.org/plosone/s/file?id=ba62/PLOSOne_formatting_sample_title_authors_affiliations.pdf> | We thank the Editor for this reminder. We have revised the manuscript to ensure full compliance with PLOS ONE style and formatting requirements, including correct file naming, and have used the provided templates. |
| Thank you for stating the following in the Acknowledgments Section of your manuscript:  “We acknowledge the participants and the administrators of Gandhi Memorial Hospital and Zewditu Memorial Hospital for their collaboration. We also acknowledge funding support from the Vanier Canada Graduate Scholarship and the International Development Research Centre (IDRC) Doctoral Research Award. Institutional support from Debre Berhan University and the University of Alberta is gratefully recognized.”  We note that you have provided additional information within the Acknowledgements Section that is not currently declared in your Funding Statement. Please note that funding information should not appear in the Acknowledgments section or other areas of your manuscript. We will only publish funding information present in the Funding Statement section of the online submission form. | Thank you for this clarification. The support received was in the form of a scholarship and award, and no specific funding number is associated with it. The funding information has been reported accordingly in the Funding Statement, and it has been removed from the Acknowledgments section in line with PLOS guidelines. We have also deleted in the submission portal. |
| Please remove any funding-related text from the manuscript and let us know how you would like to update your Funding Statement. Currently, your Funding Statement reads as follows:  “YAA received the Vanier Canada Graduate Scholarship (Government of Canada; <https://vanier.gc.ca> ) and the IDRC Doctoral Research Award (International Development Research Centre; <https://idrc-crdi.ca> ). The funders had no role in study design, data collection and analysis, decision to publish, or preparation of the manuscript”  Please include your amended statements within your cover letter; we will change the online submission form on your behalf. | We thank the Editor for this clarification. We have removed all funding-related text from the Acknowledgments section. The corrected Funding Statement is provided in the cover letter, and we kindly request that the online submission form be updated accordingly. |
| We note that the grant information you provided in the ‘Funding Information’ and ‘Financial Disclosure’ sections do not match. When you resubmit, please ensure that you provide the correct grant numbers for the awards you received for your study in the ‘Funding Information’ section | Thank you for this comment. The support received for this study was in the form of a scholarship and an award, and no grant numbers were assigned. We have revised the funding information to ensure consistency between the Funding Information and Financial Disclosure sections. |
| Thank you for stating the following in your Competing Interests section:  “NO authors have competing interests”  Please complete your Competing Interests on the online submission form to state any Competing Interests. If you have no competing interests, please state "The authors have declared that no competing interests exist.", as detailed online in our guide for authors at <http://journals.plos.org/plosone/s/submit-now>  This information should be included in your cover letter; we will change the online submission form on your behalf. | We thank you for this clarification. We confirm that the correct statement is: “The authors have declared that no competing interests exist.” This has been included in the cover letter, and we kindly request that the online submission form be updated accordingly. |
| PLOS requires an ORCID iD for the corresponding author in Editorial Manager on papers submitted after December 6th, 2016. Please ensure that you have an ORCID iD and that it is validated in Editorial Manager. | Thank you for the reminder. The corresponding author’s ORCID iD has been provided and validated in Editorial Manager. |
| We note that you have indicated that there are restrictions to data sharing for this study. PLOS only allows data to be available upon request if there are legal or ethical restrictions on sharing data publicly.  Before we proceed with your manuscript, please address the following prompts:  a) If there are ethical or legal restrictions on sharing a de-identified data set, please explain them in detail.  b) If there are no restrictions, please upload the minimal anonymized data set necessary to replicate your study findings. | Thank you for the comment. All relevant data are within the paper and its Supporting Information file. |
| Please include captions for your Supporting Information files at the end of your manuscript, and update any in-text citations to match accordingly. | Thank you for the reminder. Supporting Information captions and in-text citations have been updated. See at the end of the document in the revised file. |
| If the reviewer comments include a recommendation to cite specific previously published works, please review and evaluate these publications to determine whether they are relevant and should be cited. | Thank you for this comment. We have carefully reviewed the suggested publications and cited those that are relevant to the scope and context of our study. |
| **Additional Editor Comments** | |
| **Page 16 (Study Setting)**  The authors should briefly clarify the rationale for selecting only two hospitals in Addis Ababa and how these sites were expected to provide diverse perspectives. | Thank you for this comment. We have clarified in the Methods section the rationale for selecting the two hospitals and how they were expected to capture diverse perspectives. |
| **Study conducted only in Addis Ababa**  The study was conducted exclusively in Addis Ababa, Ethiopia’s largest and most well resourced city. Individuals in smaller or peripheral urban areas may have different experiences with PCC due to variations in resources, cultural norms, and health system access. | Thank you for this important comment. The Limitations section has been revised to note that, consistent with qualitative research, and as the study was conducted in Addis Ababa, transferability to other urban Ethiopian settings with different resources and contexts should be interpreted with caution. |
| **Page 17 (Data Collection)**  The manuscript states that: “Drafts were pilot tested for clarity and cultural appropriateness before use.” it is unclear how the pilot testing was conducted.  analysis. | Thank you for the comment. The interview guides were pilot tested with three participants similar to the study population, who were not included in the final sample. Please see the Methods section for details. |
| **Page 19**  The authors state that information power guided data collection, which is appropriate for Interpretive Description. However, the manuscript does not explain how sufficient information power was determined. | Thank you for the comment. Sufficient information power was assessed based on the study aim, sample specificity, the depth and richness of participants’ narratives, and the interpretive description analytic approach. Please see the revised Methods section for clarification. |
| **Page 21 (Overview of Findings)**  The results section is rich and insightful; however, it is excessively detailed. | Thank you for this suggestion. We have shortened the narrative and moved some longer quotations to Supporting Information to improve readability**. see Supplementary File 4.** |
| **Discussion**  The Discussion is strong but at times repeats results rather than synthesizing them. | Thank you for this helpful comment. The Discussion has been revised to reduce repetition, shorten dense paragraphs, and strengthen interpretation rather than restating the results. |
| Paragraph length and numbering  Some paragraphs are overly long and would benefit from being broken into shorter sections. Numbering and page numbering are needed. | Thank you for this suggestion. We have revised the manuscript by breaking long paragraphs into shorter sections and adding continuous line and page numbering throughout.. |
| **Reviewer's Responses to Questions** | |
| 1. Is the manuscript technically sound, and do the data support the conclusions? The manuscript must describe a technically sound piece of scientific research with data that supports the conclusions. Experiments must have been conducted rigorously, with appropriate controls, replication, and sample sizes. The conclusions must be drawn appropriately based on the data presented.  Reviewer #1: Yes  Reviewer #2: Yes | We thank the reviewers for confirming the technical rigor and that the data support the conclusions. |
| 2. Has the statistical analysis been performed appropriately and rigorously?  Reviewer #1: N/A  Reviewer #2: Yes | Thank you for your feedback. |
| 3. Have the authors made all data underlying the findings in their manuscript fully available?  The [PLOS Data policy](http://track.editorialmanager.com/CL0/http:%2F%2Fwww.plosone.org%2Fstatic%2Fpolicies.action%23sharing/1/010f019c6ee47620-1923a5d5-b20a-4c67-820f-05c2369cf6bd-000000/DW9TACWkIz8WCZ1k93QAj9CiVyqMTSVpqDPpzK_AkxU=251) requires authors to make all data underlying the findings described in their manuscript fully available without restriction, with rare exception (please refer to the Data Availability Statement in the manuscript PDF file). The data should be provided as part of the manuscript or its supporting information, or deposited to a public repository. For example, in addition to summary statistics, the data points behind means, medians and variance measures should be available. If there are restrictions on publicly sharing data—e.g. participant privacy or use of data from a third party—those must be specified.  Reviewer #1: No  Reviewer #2: Yes | Thank you for the feedback. We have now fully complied with the PLOS Data policy. All underlying data have been provided as Supporting Information, and the Data Availability Statement has been updated accordingly. |
| 4. Is the manuscript presented in an intelligible fashion and written in standard English? PLOS ONE does not copyedit accepted manuscripts, so the language in submitted articles must be clear, correct, and unambiguous. Any typographical or grammatical errors should be corrected at revision, so please note any specific errors here.  Reviewer #1: Yes  Reviewer #2: No | Thank you for your feedback. The manuscript has been thoroughly revised to improve grammar, clarity, and overall readability. |
| **Review 1 Comments to the Author** | |
| **General comments** | |
| The manuscript addresses important issues that could contribute to / have implications for healthcare services in Ethiopia. The manuscript is well written overall. The content, introduction, and discussion sections are clearly and effectively articulated. However, the Results section requires substantial revision and reanalysis. The themes and subthemes should be more clearly derived from / aligned with the specific research question(s). | Thank you for this constructive comment. The Results section has been revised to ensure that the themes and subthemes clearly align with the study's research questions. The coding framework and thematic structure were refined using an inductive interpretive description approach, and the findings are now presented through clearer themes and a more cohesive narrative, supported by representative quotations.  Please see the color change in the revised document |
| The document does not include line numbers, which would be helpful for reviewers to refer to specific lines precisely. | Thank you for noting this. Line numbers have now been added throughout the manuscript. |
| Please provide/attach the interview guide (or topic guide) used in the study. This is important for assessing how the emerged categories and subcategories in the results relate to the questions asked. | Thank you for this comment. The interview guide has now been included as a supplementary file (**Supplementary File 2**). |
| Please ensure the manuscript matches the formatting guidelines of PLOS ONE | We thank the reviewer for this comment. We confirm that the manuscript and overall document are aligned with the PLOS ONE formatting guidelines. |
| I suggested the title should be modified as…” Exploring Knowledge, Attitudes, and Practices Regarding Preconception Care Among Reproductive-Age Individuals in Ethiopia: An Interpretive Description Study Using the Socio-Ecological and Intersectional Models” | Thank you for the helpful suggestions. After careful consideration, we adopted the title suggested by Reviewer 2, as it better aligns with the study aim and research questions while improving clarity and conciseness. The revised title is: *“Perspectives on Preconception Care in Ethiopia: Social, Cultural, and Structural Determinants.”* |
| **Abstract** |  |
| You said that in Ethiopia, PCC remains almost absent in everyday health practice; the pooled  utilization of PCC in Ethiopia is 26%. “The magnitude of preconception care utilization  and associated factors among women in Ethiopia: systematic review and meta-analysis,  2024 \| BMC Pregnancy and Childbirth”, can we say almost absent? Please justify.Similarly, you mentioned again: “This study provides the first in-depth, urban Ethiopian analysis showing how adults actively negotiate preconception care (PCC) within intersecting gender, moral, and structural systems.” However, more than three published qualitative studies on preconception care already exist in Ethiopia. As a researcher, particularly a qualitative one, honesty is essential to maintain the trustworthiness and credibility of the study | Thank you for this important comment. We have revised the wording to reflect that PCC in Ethiopia is limited and inconsistently integrated into routine health services rather than “almost absent.” We have also corrected the statement to acknowledge existing studies and clarify the specific contribution of our study. |
| You employed maximum variation sampling, yet your study participants were drawn from the reproductive-age group (both women and men), which constitutes a relatively homogeneous population. Do you believe maximum variation sampling is appropriate for this study?. | Thank you for this comment. Although participants were within the reproductive-age group, maximum variation sampling was used to capture diversity across intersecting factors such as gender, age, marital status, education, and reproductive experiences. This enabled exploration of both variability and shared patterns, consistent with the interpretive description approach. The Results were interpreted with attention to these differences and commonalities, and this has now been clarified in the Methods section. |
| Is interpretive description design more dominant in exploratory studies Or Explanatory studies? Make sure that you use the appropriate design for your research. | Thank you for this comment. Interpretive description is commonly used in exploratory qualitative research to generate practice-relevant insights from participants’ experiences. Our study aims to explore how reproductive-age individuals understand and experience preconception care within their social and health system context. Therefore, the use of interpretive description is appropriate for the exploratory nature of the research, and this has been clarified in the revised document |
| Are all the listed keywords scientifically appropriate and aligned with standard terminology? Please modify the words included in the keyword | We thank the reviewer for this helpful comment. We have revised the keywords to ensure alignment with standard scientific terminology and indexing terms. Kindly see the color change in the main document. |
| **Introduction:** |  |
| What is the primary objective of this study—to explore the knowledge, attitudes, and practices (KAP) regarding PCC, or to examine participants’ perspectives on PCC within socio-ecological and intersectional contexts? Please justify your response by referring to the study title and conclusion, as there appears to be a potential mismatch between the stated aim (KAP-focused) and the interpretive, contextual emphasis in the title and conclusion. | Thank you for this important observation. The primary objective of the study is to explore reproductive-age individuals’ perspectives on preconception care within their social and structural contexts. While knowledge, attitudes, and practices were examined as part of participants’ experiences, the analysis focused on interpreting these within socio-ecological and intersectional contexts. The aim has now been clarified in the manuscript to ensure consistency with the title, analytic approach, and conclusions. |
| **Methods** | |
| Please attach the written informed consent document.  The guides explored knowledge, attitudes, and practices regarding PCC; perceived  benefits and barriers; gender and cultural dynamics; and experiences with healthcare  providers. | Thank you for this comment. The written informed consent and interview guide documents have now been attached as supplementary files **(See S1 text and S2 text)** |
| **Result** | |
| You mentioned that pilot tests were conducted for clarity and cultural appropriateness before use. Could you elaborate on the number of participants included in the pilot, and was this pilot data included in the formal analysis? Please describe this process in detail within the data collection section. | Thank you for the comment. The pilot test included three participants similar to the study population, and these pilot interviews were not included in the final analysis. Minor refinements to wording, question order, and prompts were made based on feedback to enhance clarity and cultural appropriateness. Further details are provided in the Data Collection section of the main manuscript. |
| Five women who were planning to conceive were included. How were these women identified? What method or tool was used for their identification?. | We thank the reviewer for this important question. Women who were planning to conceive were identified in collaboration with healthcare providers working in family planning, abortion care, cervical screening, and infertility treatment units. Healthcare providers informed eligible women about the study based on their expressed pregnancy intentions during routine care, and those who indicated they were planning to conceive and were interested were then approached by the research team. This has been clarified in the Methods section |
| Detailed descriptions within each theme and subtheme are vital for rigorous analysis and reporting. | We thank the reviewer for this valuable comment. Consistent with interpretive description principles, we have presented the findings using cohesive narration to ensure that each theme and subtheme is clearly described and meaningfully connected to participants’ experiences. This approach highlights patterns, variations, and contextual influences and strengthens the rigor and interpretive depth of the analysis. See the revised document |
| The sub-themes within the seven main themes should be rewritten to consolidate similar concepts. | We thank the reviewer for this important suggestion. We have carefully reviewed the subthemes and revised the results section to consolidate overlapping and closely related concepts for greater clarity and coherence. While maintaining the interpretive description approach and preserving the contextual depth of participants’ experiences, we refined the organization and wording of subthemes to reduce redundancy and improve alignment with the main themes and research questions. These revisions are reflected in the updated Results section. See the color change in the manuscript and the illustrative participant quotations supporting thematic findings  in **Supplementary File 4** |
| Since the main purpose of using maximum variation sampling is to collect information from a heterogeneous participant group, the analysis should describe and compare the differences and similarities among participants within each theme and subtheme. | We thank the reviewer for this important comment. Consistent with maximum variation sampling and interpretive description, the analysis was conducted to identify both shared patterns and variations across participants with different genders, ages, social backgrounds, and reproductive backgrounds as described before. The findings are presented using cohesive narration to reflect commonalities as well as contextual differences within each theme. We have further clarified this in the manuscript to strengthen the interpretive depth and rigor. |
| Please specify which conceptual framework or model (e.g., the socio-ecological model) guided this study. The results section should then discuss the main findings in relation to this chosen framework. | We thank the reviewer for this important comment. Consistent with interpretive description, the analysis was conducted inductively using thematic analysis informed by ground theory approach, such as open, axial, and selective coding without imposing a predefined theoretical framework, thereby minimizing theoretical baggage. Conceptual frameworks, specifically the socio-ecological model and intersectionality, were used to inform the development of the semi-structured data collection tools and to support interpretation of the findings. This approach aligns with Thorne’s guidance that interpretive description can be informed by relevant theoretical perspectives without allowing them to predetermine the analytic process. We have clarified this and strengthened the linkage between the findings and these frameworks in the revised manuscript. |
| The overall results presentation seems shallow, incoherent, and vague. The data likely requires re-analysis, and the presentation of themes and subthemes needs to be updated accordingly. | Thank you for this comment. The analysis was conducted rigorously using iterative coding, constant comparison, and team discussions consistent with the interpretive description approach. The findings are presented through cohesive interpretive narration to capture patterns and contextual meanings. The Results section has been substantially revised, with a refined coding framework and clearer thematic structure aligned with the research questions, supported by illustrative quotations to strengthen clarity and depth. |
| **Discussion** | |
| Is the word "must" commonly used to formulate recommendations for different actors based on the findings of a single study? | Thank you for this comment. The term “must” has been revised to more appropriate wording (e.g., “should” or “may consider”) to better reflect recommendations based on a single study. |
| You state, "Policy: Ethiopia’s reproductive health policy should integrate PCC into public services with clear guidelines." However, a national preconception care guideline was developed in 2024. What is the intended implication of your recommendation, given this existing policy? | Thank you for this comment. We acknowledge that a national preconception care guideline was developed in 2024. Our recommendation refers to strengthening its implementation and integration within routine health services. As part of the larger project, we have also evaluated this guideline from the perspectives of healthcare workers and stakeholders, including managers and policymakers, and a separate manuscript is currently under preparation. |
| **Conclusion** |  |
| In your conclusion, you stated, “PCC should combine biomedical prevention with relational and gender-sensitive practice.” This statement is unclear. According to the Ethiopian PCC guideline, the definition of PCC already includes a bundle of interventions aimed at identifying and modifying biomedical, behavioral, and social risks to improve pregnancy outcomes. Why, then, does your conclusion suggest that PCC should combine biomedical prevention? The guideline already encompasses this approach. | Thank you for this insightful comment. We acknowledge that the guideline already encompasses biomedical, behavioral, and social components, even if these are not always explicitly articulated. Our intention was not to suggest the addition of biomedical prevention, but rather to highlight the importance of strengthening the practical integration of relational and gender-sensitive approaches within the delivery of PCC based on our findings. The conclusion has been revised accordingly to clarify this point and to better reflect the existing scope of the guideline while emphasizing opportunities to enhance its implementation. |
| Similarly, in your conclusion, you wrote, “Providers must engage men and women as clients and supporters while ensuring confidential, stigma-free access for adolescents and unmarried women.” According to the Ethiopian PCC guideline, couple-based services are included as a guiding principle. Your statement should more directly align with this principle of couple-centered care. | Thank you for this comment. While the guideline promotes a couple-centered approach, findings from this study and our related research with healthcare workers and stakeholders (under preparation) suggest limited clarity on how couples should be practically engaged, with few explicit provisions for men’s involvement. Participants also reported gaps in implementation, particularly for men, adolescents, and unmarried women. The conclusion has been revised accordingly. |
| Therefore, your implications and conclusions should be updated to reflect the context of the Ethiopian national PCC guideline. | Thank you for this important comment. The manuscript has been revised to better acknowledge the existing guidelines while maintaining the study’s data-driven conclusions. |
| **Reviewer 2 Comments to the Author** | |
| **Title Clarity and Focus** | |
| The title effectively captures the essence of the study but consider making it more concise by removing less critical phrases. For example, "Understanding Reproductive-Age Individuals' Perspectives on Preconception Care in Ethiopia" could be shortened to "Perspectives on Preconception Care in Ethiopia: Social, Cultural, and Structural Determinants." This focuses the reader's attention on the primary research topic. | We thank the reviewer for this helpful suggestion. We agree that a more concise title improves clarity and focus. Accordingly, we have revised the title as *“Perspectives on Preconception Care in Ethiopia: Social, Cultural, and Structural Determinants.”* |
| **Establishing Context** | |
| The background provides essential context, particularly regarding the introduction of the PCC guidelines. However, consider including a brief mention of the specific health outcomes or public health significance associated with improved PCC in Ethiopia. This might enhance the urgency and importance of the study | Thank you for this feedback. We have revised the background accordingly. |
| **Methodological Rigor** | |
| The methods section outlines an appropriate approach for this type of qualitative research. It may be beneficial to elaborate on the selection criteria for participants and the rationale for employing maximum-variation sampling. This detail could enhance the robustness of your methodology and reassure readers about the representativeness of the sample. | We thank the reviewer for this helpful suggestion. We have expanded the Methods section to clarify the participant selection criteria and the rationale for using maximum-variation sampling to capture diverse perspectives and enhance methodological rigor. |
| **Thematic Insights** | |
| The results section highlights significant themes that demonstrate the complexity of PCC uptake. To strengthen this section, consider providing brief examples or quotes from participants to illustrate the themes, particularly regarding "marital gatekeeping" and "emotional negotiations.” Rich qualitative data enhances the credibility and relatability of the findings. | Thank you for the suggestion. We have revised the Results section to include additional illustrative participant quotations to better support and clarify the identified themes. |
| **Actionable Conclusions** | |
| The conclusions successfully address the complexities of PCC practice in Ethiopia. To further improve, consider specifying actionable recommendations based on your findings, such as potential interventions for healthcare providers, community organizations, or policymakers. Explicitly connecting these recommendations to identified themes could provide a clearer pathway for implementing the PCC guidelines effectively. | Thank you for the helpful suggestion. We have revised the conclusion to include clearer, actionable recommendations and linked them more explicitly to the key themes identified in the findings. |
| **Overall Impression** | |
| Your work presents valuable insights into preconception care in Ethiopia, addressing gaps in the current literature by focusing on social and cultural determinants. By refining the title and enhancing specific sections, you can communicate your findings more effectively and emphasize the need for comprehensive, context-aware strategies in PCC implementation. | Thank you for your encouraging and constructive feedback. We have revised the manuscript accordingly to improve clarity and better communicate the study’s contributions. |
| What specific social and cultural factors significantly influence the acceptance and uptake of preconception care among different demographic groups in urban Ethiopia? | Thank you for this question. As described in the Results, PCC uptake was influenced by marital norms, stigma toward unmarried individuals, gender roles placing responsibility mainly on women, religious beliefs, and community attitudes. Education, peer influence, and community support also shaped acceptance and engagement. |
| How do gender norms and roles affect men’s involvement in preconception care, and what strategies can be implemented to encourage their active participation in this process? | Thank you for this question. Our findings showed that gender norms often position PCC as a woman’s responsibility, limiting men’s involvement and discouraging clinic participation. Participants suggested male-friendly services, couple-based counseling, and community or media awareness to promote men’s engagement. |
| **Final author's comment** |  |
| Thank you to the reviewers for their thoughtful and constructive feedback. We have carefully revised the manuscript in response to all comments and suggestions. The revisions include clarifying the study aim and research questions, refining the Results through clearer thematic presentation, aligning the conclusions and implications with the existing guidelines, and improving transparency by adding the interview guide and consent documents. We have also refined the title and strengthened several sections to enhance clarity and coherence. We appreciate the reviewers’ insights, which have significantly improved the quality and rigor of the manuscript. | |
