# Supplementary material for: Perspectives on preconception care in Ethiopia: Social, cultural, and structural determinants
Source: PLoS One. 2026 Jun 5;21(6):e0351142. doi: 10.1371/journal.pone.0351142 (PMC13240867; doi:10.1371/journal.pone.0351142)
Supplement: Attachment — Submitted filename: my reccomendations.docx [file pone.0351142.s007.docx]

I appreciate authors for conducting the preconception-related issues, which are underexplored in the Ethiopian context.

The study addresses an important area, and the integration of social, cultural, and structural perspectives provides valuable insights. The revisions have improved overall clarity and rigor.

As I have seen in the manuscript’s submission history, the authors clearly addressed the previous concerns raised by reviewers. From my side, I didn’t see any critical issues that hinder the acceptance of the manuscript.

According to the manuscript

1. The study presents the results of original research.
2. Results reported have not been published elsewhere.
3. Analyses are performed to a high technical standard and are described in sufficient detail.
4. Conclusions are presented appropriately and are supported by the data.
5. The article is presented in an intelligible fashion and is written in standard English.
6. The research meets all applicable standards for the ethics of research integrity.
7. The article adheres to appropriate reporting guidelines and community standards for data availability

I recommend that authors address the following minor issues

1. In the data analysis section – page 8 - the authors stated that they followed ID principles for data analysis, and you provided the references, which is appreciated. But I suggest you include a summary of the ID principles you specifically used, which helps convince readers and provides a clearer understanding of the entire research process - or if mentioned, indicate where they are mentioned in a point-by-point response.
2. In the Rigor and trustworthiness section – page 10 - you stated that rigor was ensured using quality criteria specific to interpretive description. Please briefly mention those quality criteria, or if mentioned, indicate where they are mentioned in point to point response.

This manuscript addresses novel and critical issues, and considering that the authors addressed previous reviewers' comments and suggestions, this manuscript has high potential for acceptance from my side.

Hence, my decision is a **minor revision**
