# Supplementary material for: Perspectives on preconception care in Ethiopia: Social, cultural, and structural determinants
Source: PLoS One. 2026 Jun 5;21(6):e0351142. doi: 10.1371/journal.pone.0351142 (PMC13240867; doi:10.1371/journal.pone.0351142)
Supplement: Attachment — Submitted filename: Reviewers comment.docx [file pone.0351142.s009.docx]

Overall, the author's response was good; however, the issues raised—especially in the results section—were not addressed adequately. These issues still need to be responded to by the author.

1. The overall presentation of the results seems shallow, incoherent, and vague. The data likely requires reanalysis, and the presentation of the themes and subthemes needs to be updated accordingly.
2. You submitted the PARTICIPANT CONSENT FORM in Supplementary File 1, but what I require is the actual form that the participants signed. This is crucial information for establishing the trustworthiness of the overall results.
3. I still do not agree with the listed keywords. You have provided the keywords according to scientific conventions, including their order.
