# Supplementary material for: Perspectives on preconception care in Ethiopia: Social, cultural, and structural determinants
Source: PLoS One. 2026 Jun 5;21(6):e0351142. doi: 10.1371/journal.pone.0351142 (PMC13240867; doi:10.1371/journal.pone.0351142)
Supplement: Attachment — Submitted filename: Response to Reviewers-R2.docx [file pone.0351142.s010.docx]

**Authors Response**

| **Editor and Reviewer Comments** | **Comments/ Revisions Made** |
| --- | --- |
| **Editor comment** | |
| General suggestion | |
| This is a **relevant and timely qualitative study** addressing an important gap in preconception care (PCC) in Ethiopia. The manuscript is generally well-written, methodologically sound, and grounded in an appropriate qualitative framework (interpretive description). The topic aligns well with global health priorities. | Thank you, Dear Editor, for your positive and encouraging feedback. |
| **Comments** |  |
| The manuscript would benefit from a clearer and more explicit justification for the use of interpretive description (ID) over other qualitative approaches.   - While ID is appropriate for generating practice-oriented insights, the authors should clarify: - Why ID is particularly suited to addressing the study objectives - How it enables insights beyond what other qualitative designs might offer | Dear Editor,  Thank you for your feedback. We have revised the manuscript accordingly. Please also note that this paper provides only a brief introduction and methodological overview, as it is part of a larger dissertation project. The full study includes healthcare workers, stakeholders, and reproductive-age individuals, and a dedicated dissertation chapter presents detailed methods, including the rationale for selecting interpretive description over other qualitative approaches. We encourage interested readers to refer to that for a more comprehensive account in the near future . |
| - There remains a lack of conceptual clarity regarding the study’s framing. Although the manuscript aims to provide an interpretive, socio-ecological understanding of preconception care (PCC), the research questions and parts of the analysis still reflect a KAP orientation. | Thank you for this comment. We respectfully note that this was addressed in our R1 revision. The research question has been revised to focus on in-depth perspectives and experiences rather than discrete knowledge or attitudes, consistent with ID's interpretive focus. While knowledge, attitudes, and practices emerged as part of participants' experiences, the analysis interpreted these within socio-ecological and intersectional contexts. The aim has been further clarified in the revised manuscript to ensure alignment with the title, analytic approach, and conclusions; please see the highlighted changes |
| - The authors should clearly by either: - Removing explicit KAP language from the research questions, or - Explicitly justifying how KAP elements are being reinterpreted through a socio-ecological and intersectional lens - This alignment is essential to ensure coherence between the study aim, methodology, and findings. | Thank you for this comment. We respectfully note that this was addressed in our R1 revision. We have nonetheless conducted a thorough review of the manuscript to ensure full alignment; please see the highlighted changes." |
| Result |  |
| The results section, while rich, remains largely descriptive and would benefit from deeper interpretive analysis.  The manuscript should move beyond describing themes to explaining:  How and why factors interact  The mechanisms underlying observed patterns  The relationships between themes  For example, the discussion of men’s exclusion from PCC services would be strengthened by explicitly analyzing how gender norms, health service design and institutional practices interact to produce and reinforce this exclusion.  Strengthening this level of interpretation will significantly enhance the analytical contribution of the study. | Thank you for this suggestion. We have addressed it; please see the changes highlighted in the revised manuscript. |
| **Integration of Theoretical Frameworks** | |
| Although the socio-ecological model and intersectionality are mentioned, their integration into the findings is not sufficiently explicit.  The authors should clearly (individual, interpersonal, community, institutional) and explicitly demonstrate : how gender, marital status, age, and socioeconomic position interact to shape PCC access and experiences.  This will improve conceptual coherence and demonstrate how theory informs the interpretation of results. | Thank you for this comment. As clarified in the manuscript, the socio-ecological model and intersectionality informed interpretation rather than structuring the analysis a priori. We have strengthened the presentation to more explicitly demonstrate how factors across levels and intersecting identities shape PCC experiences in the findings. |
| Given that Ethiopia has already introduced a national PCC guideline (2024), the manuscript should more clearly frame its implications in terms of implementation gaps rather than suggesting the introduction of PCC.  Specifically, the authors should clarify how their findings explain why PCC remains weakly integrated into routine practice and link identified barriers to failures in implementation rather than absence of policy. This reframing will enhance the policy relevance and accuracy of the study | Thank you. We have revised it. |
| **Reviewer's Responses to Questions** | |
| 1. If the authors have adequately addressed your comments raised in a previous round of review and you feel that this manuscript is now acceptable for publication, you may indicate that here to bypass the “Comments to the Author” section, enter your conflict of interest statement in the “Confidential to Editor” section, and submit your "Accept" recommendation.  Reviewer #1: (No Response)  Reviewer #2: (No Response)  Reviewer #3: (No Response) | - |
| 2. Is the manuscript technically sound, and do the data support the conclusions? The manuscript must describe a technically sound piece of scientific research with data that supports the conclusions. Experiments must have been conducted rigorously, with appropriate controls, replication, and sample sizes. The conclusions must be drawn appropriately based on the data presented.  Reviewer #1: Yes  Reviewer #2: Yes  Reviewer #3: Yes | We thank the reviewers for confirming the technical rigor and that the data support the conclusions. |
| 3. Has the statistical analysis been performed appropriately and rigorously?  Reviewer #1: N/A  Reviewer #2: Yes  Reviewer #3:Yes | Thank you for your feedback. |
| 4. Have the authors made all data underlying the findings in their manuscript fully available?  The [PLOS Data policy](http://track.editorialmanager.com/CL0/http:%2F%2Fwww.plosone.org%2Fstatic%2Fpolicies.action%23sharing/1/010f019c6ee47620-1923a5d5-b20a-4c67-820f-05c2369cf6bd-000000/DW9TACWkIz8WCZ1k93QAj9CiVyqMTSVpqDPpzK_AkxU=251) requires authors to make all data underlying the findings described in their manuscript fully available without restriction, with rare exception (please refer to the Data Availability Statement in the manuscript PDF file). The data should be provided as part of the manuscript or its supporting information, or deposited to a public repository. For example, in addition to summary statistics, the data points behind means, medians and variance measures should be available. If there are restrictions on publicly sharing data—e.g. participant privacy or use of data from a third party—those must be specified.  Reviewer #1: No  Reviewer #2: Yes  Reviewer #3:Yes | Thank you for the feedback. We have now fully complied with the PLOS Data policy. All underlying data have been provided as Supporting Information, and the Data Availability Statement has been updated accordingly. |
| 5. Is the manuscript presented in an intelligible fashion and written in standard English? PLOS ONE does not copyedit accepted manuscripts, so the language in submitted articles must be clear, correct, and unambiguous. Any typographical or grammatical errors should be corrected at revision, so please note any specific errors here.  Reviewer #1: Yes  Reviewer #2: No  Reviewer #3:Yes | Thank you for your feedback. The manuscript has been thoroughly revised to improve grammar, clarity, and overall readability. |
| **Review Comments to the Author** | |
| **Reviewer 1 Comments to the Author** | |
| Overall, the author's response was good; however, the issues raised—especially in the results section—were not addressed adequately. These issues still need to be responded to by the author. | Thank you for your feedback. We sincerely appreciate it and have made further revisions accordingly. |
| The overall presentation of the results seems shallow, incoherent, and vague. The data likely requires reanalysis, and the presentation of the themes and subthemes needs to be updated accordingly. | Thank you for your comment. We believe the study robustly addresses the research questions and fully adheres to ID expectations. Nonetheless, to further satisfy the reviewer, we have refined the analysis and updated the themes and subthemes to improve clarity, coherence, and depth; please see the highlighted changes in the revised manuscript. |
| You submitted the PARTICIPANT CONSENT FORM in Supplementary File 1, but what I require is the actual form that the participants signed. This is crucial information for establishing the trustworthiness of the overall results | Thank you for your feedback and concern. Due to ethical and confidentiality considerations, we are unable to upload the signed participant consent forms. However, ethical approval details are provided in the manuscript, and the relevant ethics board can be contacted if verification is required. |
| I still do not agree with the listed keywords. You have provided the keywords according to scientific conventions, including their order | Thank you for your comment. We have revised it. |
| **Reviewer 2 Comments to the Author** | |
| The manuscript addresses an important topic and presents rich qualitative data, but several methodological, analytical, and reporting issues must be addressed before this paper can be considered for publication. revisions are required to clarify sampling, analytic rigor, conceptual framing, and the linkage between findings and clinical or policy implications | Dear Reviewer,  Thank you for your feedback. We have revised the manuscript to address sampling, analytic rigor, conceptual framing, and clinical and policy implications. |
| **Framing, citations, and epidemiological currency** | |
| Citation over-reliance: The manuscript appears to rely heavily on a single citation ([25]) to frame the core problem statement. Please broaden and update the literature review to include more recent and regionally relevant studies (local Ethiopian and sub-Saharan African PCC literature) to situate your contribution within the current discourse. | Thank you for this comment. We have revised it accordingly. |
| Outdated epidemiological data: The maternal mortality and other public-health statistics cited seem outdated. Please update these figures to the latest sources (e.g., recent EDHS, WHO/UNICEF 2024 updates) and justify your choice of statistics to ensure clinical and policy relevance. | Dear Reviewer,  Thank you for this comment. We have updated the epidemiological data using recent sources. |
| **Sampling, representativeness, and generalizability** | |
| Sample size and representativeness: The study was conducted in a single capitalcity setting with only 18 participants. Please clarify that findings are not nationally representative and discuss transferability limits. Avoid implying national generalizability based on this urban, hospital-based sample.  Sample composition and gender split: Provide methodological justification for the 10 women / 8 men composition. Was this ratio purposeful, convenience-based, or determined by saturation? Explain how you assessed saturation for each gender subgroup, and justify claims (e.g., “men’s latent desire”) given the small male sample. | Thank you for these points. We have addressed both concerns in the limitations section and clarified that the study assessed information power rather than data saturation, based on the study aim, sample specificity, narrative depth, and the interpretive descriptive approach. We respectfully note that these issues were addressed in detail in our R1 revision, and we kindly request that the reviewer refer to the revised manuscript before raising further concerns on these points; please see the highlighted changes for full details |
| **Methods and design choices** | |
| Choice of Interpretive Description (ID): Explain why ID was selected over other qualitative frameworks (e.g., Grounded Theory, Phenomenology). Specifically justify how the applied nature of ID produced clinically relevant insights that a traditional thematic or descriptive approach could not. | Thank you for this comment. We have clarified the rationale for selecting interpretive description. Please see the highlighted changes for full details |
| Research questions and methodological alignment: o KAP framing vs. ID: Research Question 1 is framed in KnowledgeAttitudes-Practices (KAP) terms, which aligns poorly with ID’s interpretive, practice-oriented focus. Explain why a KAP-style question was used and how it allows for the depth of interpretation expected in ID. | Thank you for this comment. We respectfully note that this issue was addressed in our R1 revision, where the research question was revised to focus on in-depth perspectives and experiences rather than discrete knowledge or attitudes, strengthening alignment with the interpretive, practice-oriented focus of ID. We kindly request that the reviewer refer to the updated manuscript; please see the highlighted changes for full details |
| Passive phrasing: RQ1’s wording (“What can be learned...”) is passive and undercuts the applied focus of ID. Consider rephrasing to emphasize processes of negotiation or construction of PCC in context. | Thank you for this comment. The research questions have been revised to ensure active wording and better alignment with the applied focus of interpretive description. |
| Clinical utility: ID prioritizes clinical relevance. Neither RQ explicitly asks about implications for service delivery, nursing, or midwifery practice. Consider adding or revising an RQ to directly probe actionable pathways for health-system adaptation. | Thank you for this suggestion. We note that this study forms part of a larger qualitative interpretive project exploring PCC across multiple groups, including healthcare workers and stakeholders, with findings collectively oriented toward actionable insights for service delivery and nursing and midwifery practice. We have revised the research questions to more explicitly reflect this; please see the highlighted changes. |
| **Analytic rigor and reflexivity** | |
| Transcription and translation: Provide details on transcription and translation processes (who transcribed/translated, whether translations were back-translated or verified, and steps taken to preserve meaning). | Dear Reviewer,  Thank you. We have clarified the transcription and translation procedures, including verification and meaning preservation. |
| Coding and analytic procedures: Describe the coding team, development and use of a codebook, procedures for inter-coder checking, and how themes were iteratively developed. If only one analyst, detail measures used to enhance credibility (peer debriefing, memoing, triangulation). | Thank you. We have clarified the coding process, team roles, and steps taken to enhance analytic rigor, including iterative development and verification procedures. |
| Saturation and thematic adequacy: Explain how you assessed sufficiency of data and reached saturation for the reported seven themes given 18 interviews. | Thank you for this comment. As addressed in R1, sample adequacy was guided by information power, and we have clarified how this supports the development of the reported themes. |
| Reflexivity: Add a reflexivity statement describing researcher positionality (interviewer gender, language skills, professional roles), relationships with participants, and how potential biases were managed. | Thank you. We have added a reflexivity statement outlining researcher positionality, relationships with participants, and steps taken to manage potential biases. |
| **Thematic presentation and conceptual clarity** | |
| Evidence and quotes: Strengthen each theme with clearer definitions and representative verbatim quotes (with consistent pseudonyms and key participant characteristics: gender, age, marital status). For strong claims (e.g., “institutional exclusion of unmarried women”), clearly delineate whether evidence reflects formal policy, provider attitudes, or actual practices. | Thank you. This has already been addressed in the revised manuscript. See supplementary file 4 |
| Agency and hidden practices: Your sentence about participants’ improvised strategies (using pharmacies discretely, social media, disguising visits) is important but currently unclear and missing a citation (“Such hidden practices echo Scott’s (33) not clear, line number”). Clarify the Scott citation (complete reference and correct line/section if quoting), and ensure the prose reads clearly (line numbers do not belong in the narrative). | Thank you. We have revised this section, clarified the description, and corrected the citation. |
| **Interpretation, alternative explanations, and reader comprehension** | |
| Interpretation vs. data support: Some interpretations extend beyond presented data. Where possible, link claims more explicitly to excerpts or examples. Consider alternative explanations for behaviors (e.g., pragmatic access barriers vs. moral objections) and discuss how you adjudicated between them. | Thank you. We have revised the manuscript to more closely link interpretations to data excerpts and have considered alternative explanations in the analysis. |
| “Latent” constructs and affective states: When describing men’s “latent desire” or participants’ emotions (fear, guilt, hope), clarify how these were captured and analyzed. Provide examples showing whether these affective states were directly voiced, inferred from narratives, or derived from field notes. Outline how the coding scheme or memoing systematically captured affect rather than relying on anecdote. | Thank you. We have clarified how these constructs were identified and analyzed, including whether they were directly expressed or inferred, and outlined how coding and memoing systematically captured these affective dimensions. |
| Biomedical risks: The manuscript references “biomedical risks” that readers (especially non-clinical social scientists) may not fully understand. Define salient biomedical risks related to PCC in the Ethiopian context and briefly explain their relevance to readers across disciplines | Dear Reviewer,  Thank you. We have revised the manuscript. |
| **Research questions and intersectionality** | |
| Misalignment between RQs and ID: Reassess the fit between KAP-focused RQ1 and an interpretive, clinical-application oriented ID approach. Either reframe RQ1 or more clearly explain how a KAP lens was interrogated interpretively. | Thank you. We have revised it |
| Operationalizing intersectionality: RQ2 lists multiple intersecting determinants (gender, SES, culture, accessibility). Describe analytic strategies used to move beyond listing factors to showing intersections (e.g., comparative case analysis, deviant case sampling, matrix coding). Provide examples illustrating how intersectional differences manifest (e.g., low-SES unmarried woman vs. high-SES married woman). | Thank you for this observation. We respectfully clarify that intersectionality and the socio-ecological model were not used as analytic frameworks during the coding and analysis process. As explicitly stated in the methods section, these frameworks informed the development of the research questions and provided a lens for the final interpretation and contextualisation of findings; please see the relevant section of the revised manuscript. |
| Scope vs. sample size: Given the small, hospital-based sample, temper claims about broad socio-ecological mapping and emphasize that findings reflect subjective experiences of these factors rather than generalizable structural mapping. | Thank you. As qualitative research does not aim for generalizability, we have clarified that the findings reflect context-specific, in-depth insights. |
| **Ethical considerations and reporting** | |
| IRB and consent: Provide IRB approval details (committee, reference number) and describe the informed consent process (written/oral), confidentiality protections, and whether participants received compensation. | Dear Reviewer,  Thank you. The ethical approval letters were already submitted as supporting documents, and details are included in the manuscript. For confidentiality reasons, this was also addressed in the cover letter in R1. |
| Sensitive topics and participant safety: Given discussions of marital gatekeeping and potential stigmatized behavior, explain procedural safeguards used to protect participants from harm or identification. | Thank you. We have clarified the procedural safeguards used to protect participants, including confidentiality, de-identification, and ethical handling of sensitive topics. |
| **Policy and practice implications** | |
| From findings to recommendations: The manuscript suggests engaging faith leaders, integrating men, and regulating pharmacies/digital platforms. Please provide concrete, feasible recommendations and potential steps for operationalizing the 2024 national PCC guideline (e.g., service redesign examples, training modules, pilot interventions, regulatory mechanisms). | Thank you for this suggestion. Our recommendations are grounded in the study findings, and we have clarified this while outlining feasible steps for implementation. We also note that our larger qualitative interpretive project includes interviews with healthcare workers and stakeholders, and the recommendations presented here are further aligned with and supported by findings from those complementary components of the project. |
| **Reviewer 3 Comments to the Author** | |
| I appreciate authors for conducting the preconception-related issues, which are underexplored in  the Ethiopian context.  The study addresses an important area, and the integration of social, cultural, and structural  perspectives provides valuable insights. The revisions have improved overall clarity and rigor.  As I have seen in the manuscript’s submission history, the authors clearly addressed the previous  concerns raised by reviewers. From my side, I didn’t see any critical issues that hinder the  acceptance of the manuscript. | Dear Reviewer,  Thank you for your encouraging feedback. We sincerely appreciate your recognition of our work and the improvements made. |
| According to the manuscript  1. The study presents the results of original research.  2. Results reported have not been published elsewhere.  3. Analyses are performed to a high technical standard and are described in sufficient detail.  4. Conclusions are presented appropriately and are supported by the data.  5. The article is presented in an intelligible fashion and is written in standard English.  6. The research meets all applicable standards for the ethics of research integrity.  7. The article adheres to appropriate reporting guidelines and community standards for data  Availability | Thank you for your assessment. |
| **Recommendation** | |
| I recommend that authors address the following minor issues  1. In the data analysis section – page 8 - the authors stated that they followed ID principles for  data analysis, and you provided the references, which is appreciated. But I suggest you  include a summary of the ID principles you specifically used, which helps convince readers  and provides a clearer understanding of the entire research process - or if mentioned, indicate  where they are mentioned in a point-by-point response. | Thank you for this critical question. This has been modified in the main document |
| In the Rigor and trustworthiness section – page 10 - you stated that rigor was ensured using  quality criteria specific to interpretive description. Please briefly mention those quality  criteria, or if mentioned, indicate where they are mentioned in point to point response.  This manuscript addresses novel and critical issues, and considering that the authors  addressed previous reviewers&#39; comments and suggestions, this manuscript has high potential  for acceptance from my side.  Hence, my decision is a minor revision | Thank you for this comment. We respectfully note that these quality criteria are already explicitly described in the rigor and trustworthiness section of the manuscript, including epistemological integrity, representative credibility, analytic logic, and interpretive authority. We kindly refer the reviewer to page 10 of the revised manuscript for full details |
