# Supplementary material for: Perspectives on preconception care in Ethiopia: Social, cultural, and structural determinants
Source: PLoS One. 2026 Jun 5;21(6):e0351142. doi: 10.1371/journal.pone.0351142 (PMC13240867; doi:10.1371/journal.pone.0351142)
Supplement: Attachment — Submitted filename: Reviewer comments.pdf [file pone.0351142.s008.pdf]

Manuscript Number PONE-D-25-65367
"Perspectives on preconception care in Ethiopia: social, cultural, and structural determinants."

Reviewer's Critical Comments and Questions

Summary impression

- The manuscript addresses an important topic and presents rich qualitative data, but several methodological, analytical, and reporting issues must be addressed before this paper can be considered for publication. Major revisions are required to clarify sampling, analytic rigor, conceptual framing, and the linkage between findings and clinical or policy implications.

1. Framing, citations, and epidemiological currency

- Citation over-reliance: The manuscript appears to rely heavily on a single citation ([25]) to frame the core problem statement. Please broaden and update the literature review to include more recent and regionally relevant studies (local Ethiopian and sub-Saharan African PCC literature) to situate your contribution within the current discourse.
- Outdated epidemiological data: The maternal mortality and other public-health statistics cited seem outdated. Please update these figures to the latest sources (e.g., recent EDHS, WHO/UNICEF 2024 updates) and justify your choice of statistics to ensure clinical and policy relevance.

2. Sampling, representativeness, and generalizability

- Sample size and representativeness: The study was conducted in a single capital-city setting with only 18 participants. Please clarify that findings are not nationally representative and discuss transferability limits. Avoid implying national generalizability based on this urban, hospital-based sample.
- Sample composition and gender split: Provide methodological justification for the 10 women / 8 men composition. Was this ratio purposeful, convenience-based, or determined by saturation? Explain how you assessed saturation for each gender subgroup, and justify claims (e.g., "men's latent desire") given the small male sample.

3. Methods and design choices

- Choice of Interpretive Description (ID): Explain why ID was selected over other qualitative frameworks (e.g., Grounded Theory, Phenomenology). Specifically justify how the applied nature of ID produced clinically relevant insights that a traditional thematic or descriptive approach could not.
- Research questions and methodological alignment:

- KAP framing vs. ID: Research Question 1 is framed in Knowledge-Attitudes-Practices (KAP) terms, which aligns poorly with ID's interpretive, practice-oriented focus. Explain why a KAP-style question was used and how it allows for the depth of interpretation expected in ID.
- Passive phrasing: RQ1's wording ("What can be learned...") is passive and undercuts the applied focus of ID. Consider rephrasing to emphasize processes of negotiation or construction of PCC in context.
- Clinical utility: ID prioritizes clinical relevance. Neither RQ explicitly asks about implications for service delivery, nursing, or midwifery practice. Consider adding or revising an RQ to directly probe actionable pathways for health-system adaptation.

4. Analytic rigor and reflexivity

- Transcription and translation: Provide details on transcription and translation processes (who transcribed/translated, whether translations were back-translated or verified, and steps taken to preserve meaning).
- Coding and analytic procedures: Describe the coding team, development and use of a codebook, procedures for inter-coder checking, and how themes were iteratively developed. If only one analyst, detail measures used to enhance credibility (peer debriefing, memoing, triangulation).
- Saturation and thematic adequacy: Explain how you assessed sufficiency of data and reached saturation for the reported seven themes given 18 interviews.
- Reflexivity: Add a reflexivity statement describing researcher positionality (interviewer gender, language skills, professional roles), relationships with participants, and how potential biases were managed.

5. Thematic presentation and conceptual clarity

- Number and coherence of themes: The manuscript reports seven themes, which risks fragmentation. Explain why seven themes are optimal rather than synthesizing into higher-order constructs. Show how you avoided thematic blurring between closely related themes (e.g., "faith-based negotiations" vs. "moral systems").
- Evidence and quotes: Strengthen each theme with clearer definitions and representative verbatim quotes (with consistent pseudonyms and key participant characteristics: gender, age, marital status). For strong claims (e.g., "institutional exclusion of unmarried women"), clearly delineate whether evidence reflects formal policy, provider attitudes, or actual practices.
- Agency and hidden practices: Your sentence about participants' improvised strategies (using pharmacies discretely, social media, disguising visits) is important but currently unclear and missing a citation ("Such hidden practices echo Scott's (33) not clear, line number"). Clarify the Scott citation (complete reference and correct line/section if quoting), and ensure the prose reads clearly (line numbers do not belong in the narrative).

6. Interpretation, alternative explanations, and reader comprehension

- Interpretation vs. data support: Some interpretations extend beyond presented data. Where possible, link claims more explicitly to excerpts or examples. Consider alternative explanations for behaviors (e.g., pragmatic access barriers vs. moral objections) and discuss how you adjudicated between them.
- "Latent" constructs and affective states: When describing men's "latent desire" or participants' emotions (fear, guilt, hope), clarify how these were captured and analyzed. Provide examples showing whether these affective states were directly voiced, inferred from narratives, or derived from field notes. Outline how the coding scheme or memoing systematically captured affect rather than relying on anecdote.
- Biomedical risks: The manuscript references "biomedical risks" that readers (especially non-clinical social scientists) may not fully understand. Define salient biomedical risks related to PCC in the Ethiopian context and briefly explain their relevance to readers across disciplines.

7. Research questions and intersectionality

- Misalignment between RQs and ID: Reassess the fit between KAP-focused RQ1 and an interpretive, clinical-application oriented ID approach. Either reframe RQ1 or more clearly explain how a KAP lens was interrogated interpretively.
- Operationalizing intersectionality: RQ2 lists multiple intersecting determinants (gender, SES, culture, accessibility). Describe analytic strategies used to move beyond listing factors to showing intersections (e.g., comparative case analysis, deviant case sampling, matrix coding). Provide examples illustrating how intersectional differences manifest (e.g., low-SES unmarried woman vs. high-SES married woman).
- Scope vs. sample size: Given the small, hospital-based sample, temper claims about broad socio-ecological mapping and emphasize that findings reflect subjective experiences of these factors rather than generalizable structural mapping.

8. Ethical considerations and reporting

- IRB and consent: Provide IRB approval details (committee, reference number) and describe the informed consent process (written/oral), confidentiality protections, and whether participants received compensation.
- Sensitive topics and participant safety: Given discussions of marital gatekeeping and potential stigmatized behavior, explain procedural safeguards used to protect participants from harm or identification.

9. Policy and practice implications

- From findings to recommendations: The manuscript suggests engaging faith leaders, integrating men, and regulating pharmacies/digital platforms. Please

provide concrete, feasible recommendations and potential steps for operationalizing the 2024 national PCC guideline (e.g., service redesign examples, training modules, pilot interventions, regulatory mechanisms).
